# ﻿*Bulbophyllumromklaoense* (Orchidaceae), a new species from Thailand

**DOI:** 10.3897/phytokeys.238.114999

**Published:** 2024-02-20

**Authors:** Nicha Thawara, Thitiporn Pingyot, Piyakaset Suksathan, Saroj Ruchisansakun

**Affiliations:** 1 M.Sc. Programme in Plant Science, Faculty of Graduate Studies, Mahidol University, Nakhon Pathom 73170, Thailand; 2 Department of Plant Science, Faculty of Science, Mahidol University, Bangkok 10400, Thailand; 3 Department of Pharmaceutical Botany, Faculty of Pharmacy, Mahidol University, Bangkok 10400, Thailand; 4 Queen Sirikit Botanic Garden, Mae Rim, Chiang Mai 50180, Thailand

**Keywords:** *Bulbophyllum* sect. *Lemniscata*, critically endangered, Epidendroideae, Phitsanulok Province, Southeast Asia

## Abstract

*Bulbophyllumromklaoense* (B.sect.Lemniscata) from northern Thailand is described and illustrated as a species new to science. It is most similar to *B.muscarirubrum* and *B.triste*, but differs by having inflorescences with only 4–6 reddish-brown flowers, falcate-subovate lateral sepals that are connate only in the upper half along the interior margins, petals with erose to fimbriate margins and a lip with long cilia in the distal half on the lower surface. A comparison with other similar species in the section, as well as notes on ecology, phenology, conservation assessment and a key to B.sect.Lemniscata in Thailand are also provided.

## ﻿Introduction

*Bulbophyllum* Thouars is the largest genus in Orchidaceae, encompassing approximately 2170 accepted species (POWO 2023). This mega-genus is characterised by a rhizome with 1- or 2-leaved pseudobulbs, an inflorescence that arises from the base of the pseudobulb, a mostly moveable lip attached to a distinct column foot and usually (2‒)4 often unequal waxy pollinia (Vermeulen et al. 2014a). The genus is widely distributed in tropical to subtropical regions throughout America, Africa, Asia and Australia (Dressler 1993; Vermeulen et al. 2014a). In Thailand, about 163 species have been recorded, including five new species and a new record published in the last decade (Seidenfaden 1979, 1995; Chayamarit et al. 2014; Vermeulen et al. 2014b, 2017, 2021; Pingyot et al. 2019).

Ban Romklao Botanic Garden (BRBG), a satellite garden of Queen Sirikit Botanic Garden in Chiang Mai (QSBG), was established in 1999 in Ban Romklao, Chat Trakan District, Phitsanulok Province, under the royal initiative. The garden covers an area of approximately 222 hectares, situated at an elevation of between 750 and 1300 m. It encompasses three distinct natural vegetation types: dry evergreen forest, mixed deciduous forest and lower montane forest. Adjacent to BRBG in the north and west is Phu Soi Dao National Park where Thailand’s highest sandstone mountain (2100 m a.s.l.) is found. In 2007, Mr. Nawin Inthakul, a living collection keeper, discovered a small *Bulbophyllum* on an oak tree in the lower montane forest of BRBG during his routine native plant check listing and collected some material. The living specimens were brought to the nearby BRBG orchid nursery, where they bloomed in February 2008. Subsequently, specimens were sent to the authors of the present paper for identification. However, they could not match them with any known species and, therefore, interpreted them as the representative of a new species, which is described in this article.

## ﻿Material and methods

The unknown *Bulbophyllum* specimens were collected in BRBG in Phitsanulok Province and both living and alcohol material were sent to QSBG in Chiang Mai. Alcohol material was preserved in 70% ethanol. The living plants were transplanted into an orchid ex-situ collection at the QSBG nursery, while the alcohol specimen was deposited in the Herbarium (QBG). For morphological examinations, dissections and measurements, a stereomicroscope was employed. The key to species of B.sect.Lemniscata in Thailand was drafted, based on the keys to B.sect.Tripudianthes and B.sect.Pleiophyllus in Seidenfaden (1979).

## ﻿Taxonomy

### ﻿ Bulbophyllum romklaoense

Taxon classificationPlantaeAsparagalesOrchidaceae

Pingyot & Thawara sp. nov.

49FED916-EF4C-594A-82C4-5A595F70434F

urn:lsid:ipni.org:names:77336764-1

Figs 1 , 2 , 3

#### Diagnosis.

*Bulbophyllumromklaoense* resembles *B.muscarirubrum* Seidenf. and *B.triste* Rchb.f. *Bulbophyllumromklaoense* differs from both by having 4–6-flowered inflorescences (vs. 10–24(–50)-flowered inflorescences in *B.muscarirubrum* and *B.triste*), falcate-subovate lateral sepals (vs. narrowly ovate lateral sepals in *B.muscarirubrum* and *B.triste*), petals with erose to fimbriate margins (vs. petals with ± entire margins in *B.muscarirubrum* and *B.triste*) and a lip with long cilia in the distal half on the lower surface (vs. lip entirely glabrous in *B.muscarirubrum* and *B.triste*). *Bulbophyllumromklaoense* also differs from *B.triste* by having a peduncle which is about as long as the rachis (vs. peduncle longer than twice as long as the rachis in *B.triste*).

**Figure 1.**
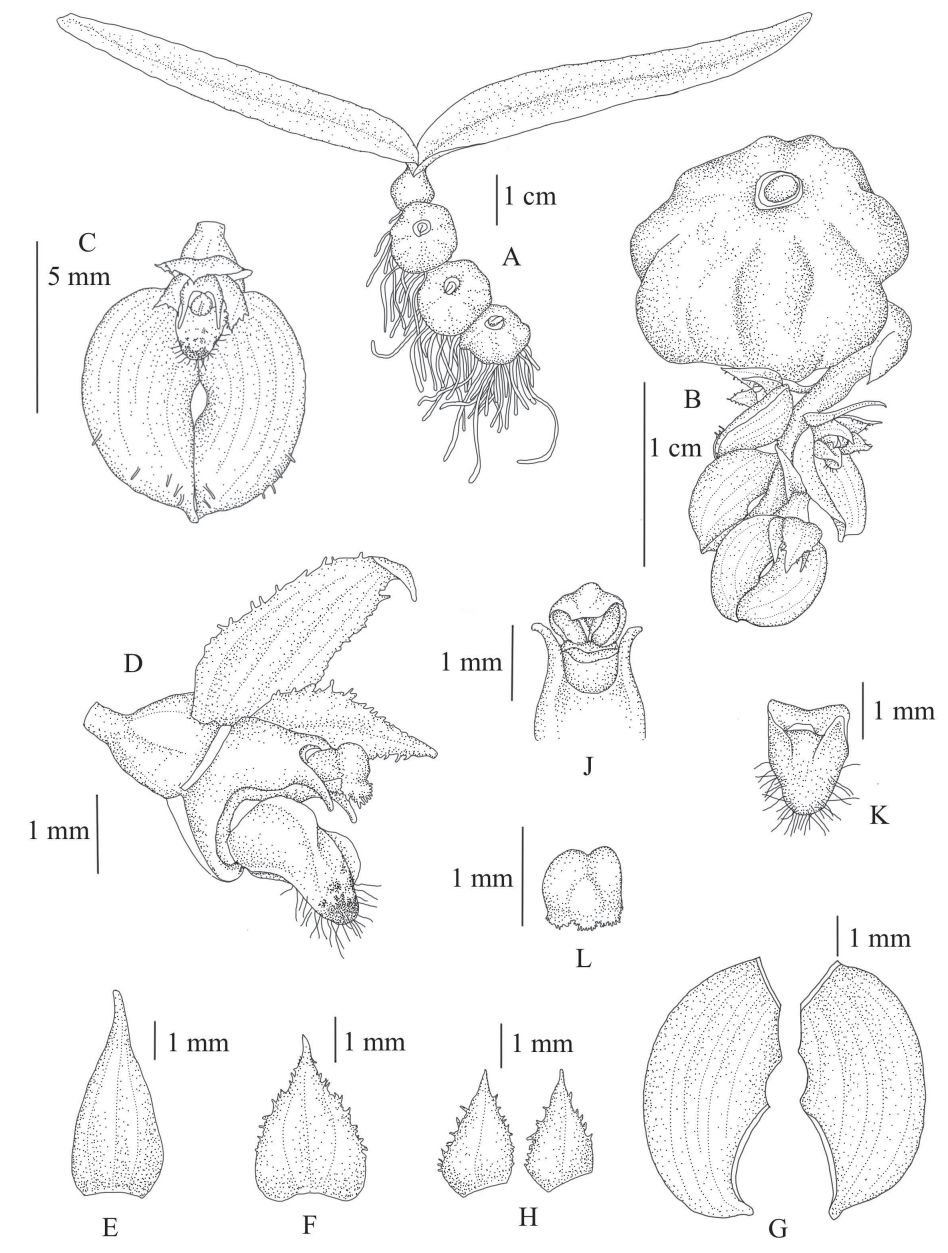
*Bulbophyllumromklaoense* Pingyot & Thawara **A** habit **B** pseudobulb with inflorescence arising from the base **C** flower, front view **D** flower, side view (right petal and right lateral sepal removed) **E** floral bract **F** dorsal sepal **G** lateral sepals (flattened & indumentum removed) **H** petals **J** column, top part **K** lip **L** anther cap (from *Inthakul* N887-50). Drawn by T. Pingyot.

#### Type.

Thailand. Phitsanulok Province, Chat Trakan District, Ban Romklao Botanic Garden, ca. 1300 m a.s.l., 15 February 2008, *Inthakul N887-50* (holotype QBG!, isotypes QBG! (2 sheets)).

#### Description.

Epiphyte with short rhizome and pseudobulbs close together. ***Pseudobulbs*** subglobose, surface slightly bullate, 10.5–25 mm in diameter, 2-leaved, pale green to purplish-green, covered with a thin and translucent-white sheath when young. ***Leaves*** shed at flowering time, narrowly ovate to oblong, 3.3–8 cm long, 0.7–1 cm wide, apex acute, base cuneate, thinly herbaceous, glabrous. ***Inflorescences*** arising from base of pseudobulb, ca. 2 cm long, prostrate, racemose, 4–6-flowered, flowers in the same inflorescence open simultaneously; peduncle 8–11 mm long, ca. 1 mm in diam., with one peduncle-scale; rachis ca. 10 mm long; floral bracts reddish, broadly lanceolate, 3.5–5.6 mm long, 1.5–2.3 mm wide, 3-veined, apex acuminate, margins entire. ***Flowers*** ca. 6 mm wide; ovary ca. 1.6 mm long, ca. 2 mm in diam., pedicel very short, inconspicuous. ***Sepals*** greenish-yellow with dense reddish-purple-brown dots especially in upper half; dorsal sepal broadly ovate, 3.7–4 mm long, 2.4–3 mm wide, apex acuminate, margins erose to fimbriate in upper half, 3-veined, adaxially papillose; lateral sepals connate in upper half along interior margins, forming a suborbicular blade in outline, individual sepals falcate-subovate, 6–6.5 mm long, 3.6–3.8 mm wide, 5-veined, adaxially sparsely ciliate in distal part, apex cuspidate, margins entire, outer margins decurved. ***Petals*** pale green with reddish-purple dots, ovate, 2.4–3 mm long, 1.7–2 mm wide, apex acuminate, margins erose to fimbriate, except near base, 1-veined, adaxially sparsely papillose and ciliate; ***lip*** white with reddish-purple dots and a large purple blotch on epichile, triangular, ca. 2 mm long, 1.3–1.5 mm wide, thickened, entire, adaxially with longitudinal ridges, with long cilia in distal half on lower surface. ***Column*** white with faint reddish-purple dots, ca. 1.5 mm long, ca. 1 mm wide, winged along lower margins; stelidia subulate, ca. 0.6 mm long, curved, pointing forwards; anther cap white, sometimes with purple marks, ca. 1 mm wide; pollinia 4; stigma concave, ca. 1 mm long. ***Fruit*** not seen.

**Figure 2.**
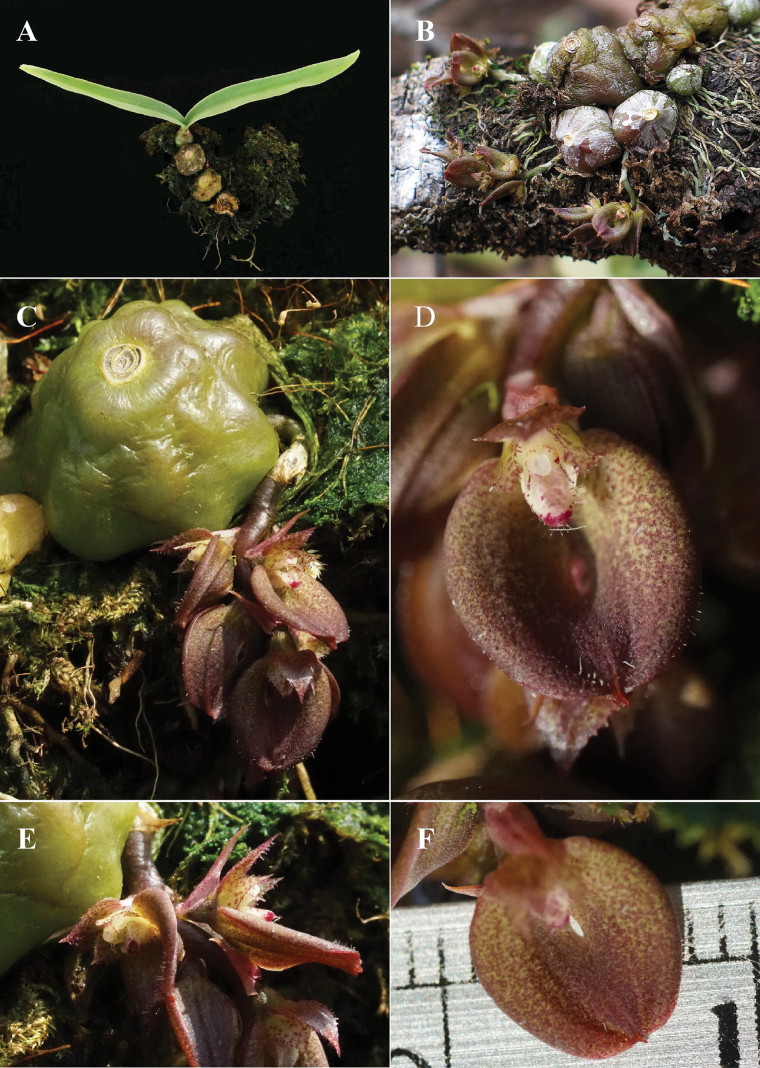
*Bulbophyllumromklaoense* Pingyot & Thawara *in vivo***A** habit (vegetative stage) **B** habit (flowering stage) **C** pseudobulb with inflorescence arising from the base **D** flower, front view **E** flowers, side view **F** lateral sepals. Photographed by P. Suksathan.

#### Habitat and phenology.

Epiphytic on oak trees (*Lithocarpus* spp.) in open evergreen broad-leaved lower montane forest, ca. 1300 m a.s.l. Fl. January–February.

#### Distribution.

**Figure 3.**
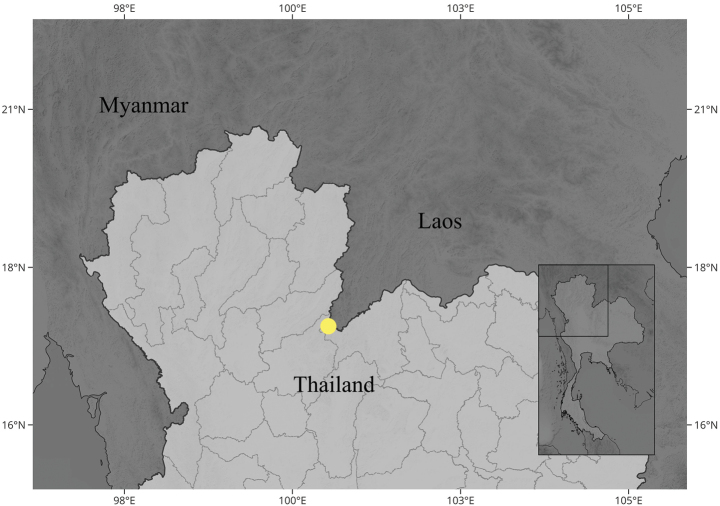
The distribution of *Bulbophyllumromklaoense* Pingyot & Thawara. The inset figure shows the position of this species on the complete map of Thailand.

Northern Thailand. This new species is currently known only from the type locality, which is located less than 7 km from the Lao PDR border. It is possible that this species occurs in Lao PDR or in other areas around the Phu Soi Dao Plateau (Fig. 3).

#### Etymology.

Named after its type locality at Ban Romklao (Romklao Village).

#### Conservation status proposed.

This new species is known only from the type locality, situated in the protected area of BRBG. However, the Extent of Occurrence (EOO) and the Area of Occupancy (AOO) are less than 100 km^2^ and 10 km^2^, respectively. The number of mature individuals is less than 50. Moreover, its habitat is frequently threatened by forest fires and climatic changes, such as warmer and drier conditions that increase drought and extend the fire season. These factors have led to significant habitat destruction. Thus, this species is preliminarily assessed as Critically Endangered (CR; B1+B2ab(iii,v)+C2a(i)), based on current information and according to the IUCN Red List Categories and Criteria (IUCN 2022).

#### Additional specimen examined.

Thailand. Phitsanulok Province, Ban Romklao Botanic Garden, ca. 1300 m a.s.l., 15 February 2008, *Inthakul N887-50* sub *Suksathan 5476* (cultivated plant of the holotype (QBG)).

#### Note.

Vermeulen et al. (2014a) redefined Bulbophyllumsect.Lemniscata Pfitz. by including B.sect.Tripudianthes Seidenf. (except *B.blepharistes* Rchb.f.) and B.sect.Pleiophyllus J.J. Sm. *Bulbophyllumromklaoense* also belongs to section Lemniscata, characterised by its two-leaved pseudobulbs, deciduous leaves, elongate racemes, 4 pollinia and connate lateral sepals. This section contains ca. 37 species, mainly distributed in South and South-East Asia (Vermeulen et al. 2014a, 2021; Averyanov et al. 2019; Zhou et al. 2022; Nguyen et al. 2023). Currently, 26 species in this section are known from Thailand. According to Seidenfaden’s key (1979), *B.romklaoense* would belong to section Pleiophyllus by its 2-leaved pseudobulb and lateral sepals that are not much longer than the dorsal sepal.

Vermeulen et al. (2014b) synonymised *Bulbophyllumtripaleum* Seidenf. under *B.dhaninivatii* Seidenf. because the only differentiating character is the presence of palea on the sepal apices, but this character is considered to be variable. We also observed this variability in a population of *B.dhaninivatii* at Phu Luang in Loei Province (north-eastern Thailand). Therefore, *B.tripaleum* is here treated as a synonym of *B.dhaninivatii* and is excluded from the key.

### ﻿Key to species of Bulbophyllumsect.Lemniscata in Thailand

**Table:** 

1	Lateral sepals with exterior margins connate	***B.polliculosum***
–	Lateral sepals free or with interior margins partially connate or connate throughout	**2**
2	Lateral sepals longer than twice as long as dorsal sepal, interior margins connate throughout their length, except near base	**3**
–	Lateral sepals up to twice as long as dorsal sepal, interior margins free or only partially connate	**13**
3	Dorsal sepal and petal apex without long thread	**4**
–	Dorsal sepal and petal apex with long thread (ca. 10 mm long), terminating in ± club-shaped tip	***B.guttifilum***
4	Dorsal sepal up to 8 mm long	**5**
–	Dorsal sepal longer than 9 mm	**12**
5	Lateral sepals with glabrous surface, rarely with a few ciliate hairs at surface or along edges; lip without globular vesicles	**6**
–	Lateral sepals with rugose-papillose surface; lip with shiny globular vesicles in upper half	***B.rugosisepalum***
6	Lateral sepals 25–55 mm long	**7**
–	Lateral sepals less than 20 mm long	***B.khaoyaiense***
7	Dorsal sepal with entire or sometimes very finely erose margins	**8**
–	Dorsal sepal with hairy-erose to distinctly erose-fimbriate margins	**10**
8	Petals with entire margins, adaxially glabrous	***B.notabilipetalum***
–	Petals with fimbriate to erose margins, adaxially papillose to hairy	**9**
9	Petals with fimbriate margins; dorsal sepal 5–6 mm long	***B.kanburiense***
–	Petals with finely erose margins; dorsal sepal 7–8 mm long	***B.dickasonii***
10	Floral bracts ovate, broadest above base	***B.tripudians***
–	Floral bracts triangular, broadest at base	**11**
11	Lip 3.4–5.5 mm long, epichile only slightly convex adaxially	***B.sphenoglossum***
–	Lip shorter, up to 3 mm long, epichile distinctly convex adaxially	***B.wallichii***
12	Dorsal sepal narrowly triangular, apex acuminate	***B.sanitii***
–	Dorsal sepal elliptic, apex obtuse to acute	***B.refractum***
13	Sepal apex with long palea; palea much longer than sepals	**14**
–	Sepal apex without or with short palea; palea if present not longer than sepals	**16**
14	Palea lamellate, with 6–10 lamellae, rectangular and radiating from an axis	**15**
–	Palea terete, finely rugose on surface	***B.lemniscatoides***
15	Inflorescence racemose, longer than 10 cm	***B.lemniscatum***
–	Inflorescence subumbellate, less than 6 cm long	***B.dhaninivatii***
16	Sepals hairy on abaxial surface	**17**
–	Sepals glabrous on abaxial surface	**21**
17	Petals ovate, margins fimbriate	***B.hirtum***
–	Petals linear, margins not fimbriate	**18**
18	Scape over 10 cm long, longer than rachis (sometimes twice as long)	**19**
–	Scape less than 1 cm long, as long as or shorter than rachis	***B.dhaninivatii***
19	Inflorescence lax-flowered; dorsal sepal 2.4–2.5 mm long	***B.reichenbachii***
–	Inflorescence dense-flowered; dorsal sepal 6 mm long or more	**20**
20	Dorsal sepal to 12 mm long; sepals with scattered long hairs on abaxial side; petals ca. 4 mm long, often twisted in upper half	***B.comosum***
–	Dorsal sepal 6–8.4 mm long; sepals with short coarse hairs on abaxial side; petals 1.6–2.6 mm long, never twisted	***B.pallidum***
21	Inflorescence lax-flowered, rachis clearly visible, flowers white to yellow	**22**
–	Inflorescence dense-flowered, rachis hardly visible, flowers purplish, reddish or brownish	**24**
22	Petal margins entire or sometimes slightly erose; ovary glabrous	***B.suavissimum***
–	Petal margins erose-serrate or fimbriate; ovary pubescent	**23**
23	Petals fimbriate along margins; dorsal sepal ca. 8.5 mm long	***B.auricomum***
–	Petals finely erose-serrate along margins; dorsal sepal ca. 5.4 mm long	***B.sukhakulii***
24	Flowering contemporary with leaves; floral bracts white, very conspicuous, ca. 10 mm long	***B.albibracteum***
–	Flowering after shedding of leaves, floral bracts not as above	**25**
25	Inflorescence 4–6-flowered; lip ciliate	***B.romklaoense***
–	Inflorescence 10–24(–50)-flowered; lip not ciliate	**26**
26	Scape much longer than rachis	***B.triste***
–	Scape as long as or shorter than rachis	***B.muscarirubrum***

## Supplementary Material

XML Treatment for Bulbophyllum romklaoense

## ﻿Additional information

### Author ORCIDs

Nicha Thawara https://orcid.org/0009-0003-6319-6741

Thitiporn Pingyot https://orcid.org/0000-0001-6917-4103

Piyakaset Suksathan https://orcid.org/0000-0003-4772-4914

Saroj Ruchisansakun https://orcid.org/0000-0002-7022-8831

### Data availability

All of the data that support the findings of this study are available in the main text.
